# Crystal structure of *trans*-bis­(di­ethano­lamine-κ^3^ *O*,*N*,*O*′)manganese(II) bis­(3-amino­benzoate)

**DOI:** 10.1107/S2056989016004072

**Published:** 2016-03-15

**Authors:** Aziz B. Ibragimov, Bakhtiyar S. Zakirov, Jamshid M. Ashurov

**Affiliations:** a Institute of General and Inorganic Chemistry of Uzbekistan Academy of Sciences, M. Ulugbek Str. 77a, Tashkent 700170, Uzbekistan; b Institute of Bioorganic Chemistry Academy of Sciences of Uzbekistan, M. Ulugbek Str. 83, Tashkent 700125, Uzbekistan

**Keywords:** crystal structure, coordination compound, 2-amino­benzoic acid, di­ethanol­amine, Mn complex, hydrogen bonding

## Abstract

Reaction of *m*-amino­benzoic acid (MABA), di­ethano­lamine (DEA) and MnCl_2_·4H_2_O led to the formation of the title salt, [Mn(C_4_H_11_NO_2_)_2_](C_7_H_6_NO_2_)_2_. In the complex cation, the Mn^2+^ ion is located on an inversion centre and is coordinated by two symmetry-related tridentate DEA mol­ecules, leading to the formation of a slightly distorted MnN_2_O_4_ octa­hedron. The MABA^−^ counter-anions are connected to the complex ion by a pair of rather strong O—H⋯O hydrogen bonds, yielding a 1:2 supra­molecular aggregate. Much weaker N—H⋯O hydrogen bonds connect neighbouring aggregates into a three-dimensional network structure.

## Abstract 2

The title salt, [Mn(C_4_H_11_NO_2_)_2_](C_7_H_6_NO_2_)_2_, contains a centrosymmetric cation with the Mn^2+^ ion coordinated octa­hedrally by two tridentate di­ethano­lamine (DEA) ligands. The cations are connected to the anions through O—H⋯O and N—H⋯O hydrogen bonds into a three-dimensional network structure.

## Chemical context   

In contrast to the two other isomers of amino­benzoic acid, *viz. p*-amino­benzoic acid (or vitamin B_10_) and *o*-amino­benzoic acid (or antranylic acid), *m*-amino­benzoic acid (3-amino­benzoic acid or MABA) is not biologically active. Nevertheless, we are studying this substance within the context of mixed-ligand coordination complex formation including benzoic acid isomers and ethano­lamines (Ashurov *et al.*, 2015 ▸). As a result of the presence of two spatially separated electron-donor functional groups in the MABA mol­ecule, the reported metal complexes of this ligand are mostly coordination polymers. Polymerization may take place involving both COOH and NH_2_ functional groups (Wang *et al.*, 2004 ▸; Flemig *et al.*, 2008 ▸; Tan *et al.*, 2006 ▸; Wei *et al.*, 2006 ▸; Shen & Lush, 2010 ▸; Wang *et al.*, 2006 ▸;), or only one of them: COOH (Kozioł *et al.*, 1992 ▸; Murugavel & Banerjee, 2003 ▸; Flemig *et al.*, 2008 ▸; Tsaryuk *et al.*, 2014 ▸) or, more infrequently, NH_2_ (Wang *et al.*, 2004 ▸).
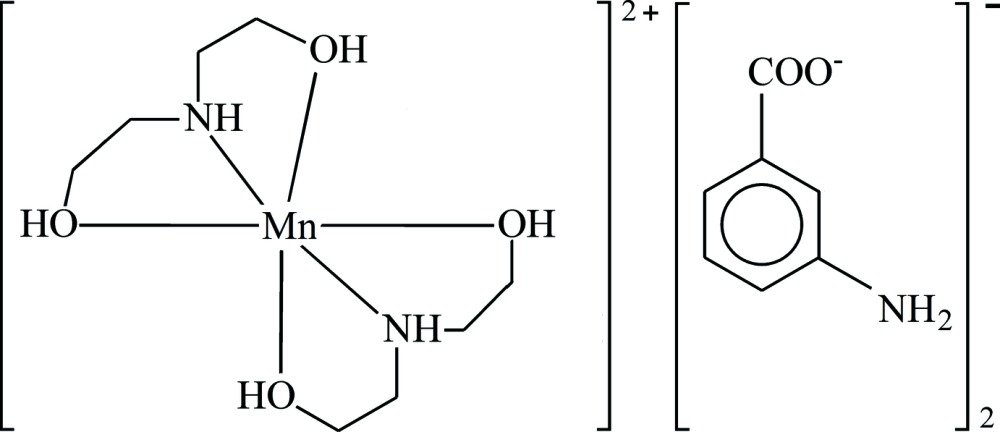


In discrete monoligand complexes, the MABA mol­ecules coordinate to metal ions only bidentately through the oxygen atoms of the carb­oxy­lic group (Ozhafarov *et al.*, 1981 ▸) while in mixed-ligand complexes, the carb­oxy­lic group can feature mono- (Sundberg *et al.*, 1998 ▸;) or bidentate (Palanisami *et al.*, 2013 ▸) coordination modes. Coordination through the nitro­gen atom is observed only in an Ag complex with participation of the co-ligand *p*-toluene­sulfonate (Smith *et al.*, 1998 ▸).

The disposition of MABA mol­ecules as non-coordinating counter-ions (in their benzoate form) is characteristic for mixed-ligand Mn (Fang & Nie, 2011 ▸) or Cd complexes (Gao *et al.*, 2011 ▸) with 4,4-bi­pyridine as co-ligand whereas the simultaneous presence of coordinating and non-coordinating MABA species was reported for an Mn complex with 1,10-phenanthroline as an additional ligand (Zhang, 2006 ▸).

Di­ethano­lamine (DEA) ligands can coordinate to metal ions in a mono- (Petrović *et al.*, 2006 ▸), bi- (Yilmaz *et al.*, 2000 ▸) or tridenentate (Buvaylo *et al.*, 2009 ▸) mode if two ligand mol­ecules are situated around the central atom. However, a combination of these modes, for example, in a bi- and tridentate fashion, is also possible (Bertrand *et al.*, 1979 ▸).

A search in the Cambridge Structural Database (CSD; Groom & Allen, 2014 ▸) revealed that crystal structures have been reported for complexes of MABA and DEA with many metal ions, including zinc, copper, nickel, manganese, cadmium, cobalt, *etc*. However, no mixed-ligand metal complex including MABA *and* DEA is documented in the CSD. In order to prepare such compounds, we carried out a synthesis in a solution containing an Mn salt, MABA and DEA. Instead of the desired complex, the title salt, [Mn(C_4_H_11_NO_2_)_2_](C_7_H_6_NO_2_)_2_, consisting of discrete [Mn(DEA)_2_]^2+^ cations and MABA^−^ anions was obtained.

## Structural commentary   

**Figure 1:**
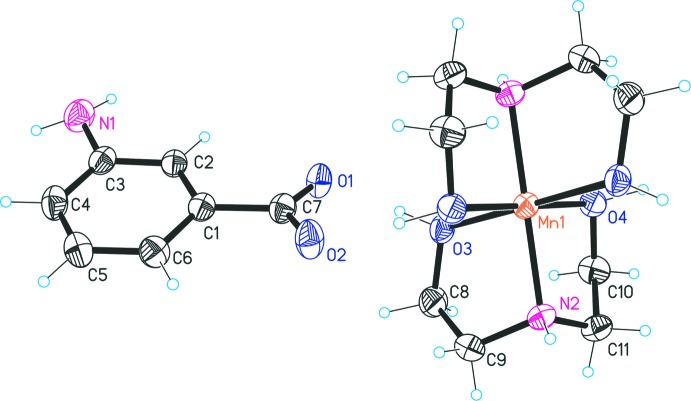
The structures of the mol­ecular moieties in the title salt. Displacement ellipsoids are drawn at the 50% probability level and the asymmetric unit is identified by the numbering of its atoms.

The asymmetric unit consists of one DEA ligand, one MABA^−^ anion and one Mn^2+^-ion, the latter being located on an inversion centre (Fig. 1 ▸). Coordination of the DEA ligand to the metal ion takes place in a tridentate *O,N,O′* mode. The Mn—ligand bond lengths cover a range from 2.065 (2) to 2.096 (2) Å with an angular range of 81.79 (10) to 98.21 (10)°, leading to a slightly distorted MnN_2_O_4_ octa­hedron. Since the DEA ligands are in their neutral form, a charged component in the outer sphere is required for charge compensation. Hence, two MABA^−^ anions in the benzoate form are present per complex ion. The carboxyl­ate group of the anionic mol­ecule is tilted by 14.4 (4)° relative to the aromatic ring.

## Supra­molecular features   

**Figure 2:**
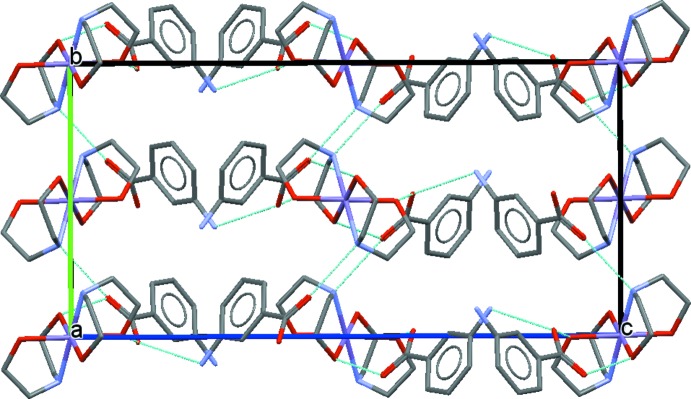
The crystal packing in the title structure. Hydrogen bonds are shown as dashed lines.

The MABA^−^ anion is connected to the complex ion by a pair of rather strong O—H⋯O hydrogen bonds involving the DEA hy­droxy groups [2.562 (3) and 2.611 (3) Å; Table 1 ▸], which give rise to the formation of a supra­molecular motif with graph-set notation 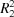
(8). The resulting supra­molecular cationic:anionic 1:2 units are associated to other such units by relatively weak N—H⋯O hydrogen bonds [2.965 (4) and 3.008 (4) Å; Table 1 ▸] involving the secondary amine function of the DEA ligand and one of the H atoms of the MABA^−^ amino group; notably, the second H atom (H1*B*) of the amino group remains without an acceptor. These four hydrogen bonds associate the different moieties into a three-dimensional network (Fig. 2 ▸).

## Synthesis and crystallization   

To an aqueous solution (5 ml) of MnCl_2_·4H_2_O (0.098 g, 0.5 mmol) was slowly added an ethano­lic solution (5 ml) containing DEA (96 µl) and MABA (0.137 g, 1 mmol) under constant stirring. A light-pink crystalline product was obtained at room temperature by solvent evaporation after 20 days.

## Refinement   

Crystal data, data collection and structure refinement details are summarized in Table 2 ▸. The positions of the O- and N-bound hydrogen atoms were located from difference Fourier maps. Whereas O-bound hydrogen atoms were refined freely, N-bound H atoms were refined with soft distance restraints of 0.98 Å for the secondary amine function and of 0.95 Å for the primary amine function. The C-bound hydrogen atoms were placed in calculated positions and refined as riding atoms with C—H = 0.93 and 0.97 Å for aromatic and methyl­ene hydrogen atoms, respectively, and with *U*_iso_(H) = 1.2*U*_eq_(C).

## Supplementary Material

Crystal structure: contains datablock(s) I. DOI: 10.1107/S2056989016004072/wm5277sup1.cif

Structure factors: contains datablock(s) I. DOI: 10.1107/S2056989016004072/wm5277Isup2.hkl

CCDC reference: 1463701

Additional supporting information:  crystallographic information; 3D view; checkCIF report

## Figures and Tables

**Table 1:** Hydrogen-bond geometry (Å, °)

*D*—H⋯*A*	*D*—H	H⋯*A*	*D*⋯*A*	*D*—H⋯*A*
N2—H2⋯O2^i^	0.96 (3)	2.19 (3)	2.965 (4)	137 (3)
N1—H1*A*⋯O1^ii^	0.97 (2)	2.05 (2)	3.008 (4)	170 (5)
O4—H4⋯O2^iii^	0.99 (5)	1.63 (5)	2.611 (3)	169 (4)
O3—H3⋯O1	0.92 (6)	1.65 (6)	2.562 (3)	173 (5)

Symmetry codes: (i) 
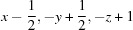
; (ii) 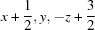; (iii) 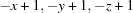.

**Table 2:** Experimental details

Crystal data	
Chemical formula	[Mn(C_4_H_11_NO_2_)_2_](C_7_H_6_NO_2_)_2_
*M*_r_	537.47
Crystal system, space group	Orthorhombic, *P* *b* *c* *a*
Temperature (K)	293
*a*, *b*, *c* (Å)	10.6120 (4), 10.8219 (4), 21.7591 (8)
*V* (Å^3^)	2498.86 (15)
*Z*	4
Radiation type	Cu *K*α
μ (mm^−1^)	4.76
Crystal size (mm)	0.32 × 0.20 × 0.18

Data collection	
Diffractometer	Oxford Diffraction Xcalibur Ruby
Absorption correction	Multi-scan (*CrysAlis PRO*; Oxford Diffraction, 2009 ▸)
*T*_min_, *T*_max_	0.932, 1.000
No. of measured, independent and observed [*I* > 2σ(*I*)] reflections	10631, 2589, 1740
*R*_int_	0.056
(sin θ/λ)_max_ (Å^−1^)	0.630

Refinement	
*R*[*F* ^2^ > 2σ(*F* ^2^)], *wR*(*F* ^2^), *S*	0.045, 0.136, 1.06
No. of reflections	2589
No. of parameters	180
No. of restraints	3
H-atom treatment	H atoms treated by a mixture of independent and constrained refinement
Δρ_max_, Δρ_min_ (e Å^−3^)	0.37, −0.22

Computer programs: *CrysAlis PRO* (Oxford Diffraction, 2009 ▸), *SHELXS97*, *XP* and *SHELXTL* (Sheldrick, 2008 ▸), *SHELXL2014* (Sheldrick, 2015 ▸) and *Mercury* (Macrae *et al.*, 2006 ▸).

## supplementary crystallographic information

### Crystal data

**Table:**

[Mn(C_4_H_11_NO_2_)_2_](C_7_H_6_NO_2_)_2_	*D*_x_ = 1.429 Mg m^−^^3^
*M**_r_* = 537.47	Cu *K*α radiation, λ = 1.54184 Å
Orthorhombic, *P**b**c**a*	Cell parameters from 1995 reflections
*a* = 10.6120 (4) Å	θ = 4.1–75.0°
*b* = 10.8219 (4) Å	µ = 4.76 mm^−^^1^
*c* = 21.7591 (8) Å	*T* = 293 K
*V* = 2498.86 (15) Å^3^	Block, pink
*Z* = 4	0.32 × 0.20 × 0.18 mm
*F*(000) = 1132	

### Data collection

**Table:**

Oxford Diffraction Xcalibur Ruby diffractometer	2589 independent reflections
Radiation source: fine-focus sealed X-ray tube	1740 reflections with *I* > 2σ(*I*)
Detector resolution: 10.2576 pixels mm^-1^	*R*_int_ = 0.056
ω scans	θ_max_ = 76.3°, θ_min_ = 4.1°
Absorption correction: multi-scan (*CrysAlis PRO*; Oxford Diffraction, 2009)	*h* = −13→11
*T*_min_ = 0.932, *T*_max_ = 1.000	*k* = −10→13
10631 measured reflections	*l* = −23→27

### Refinement

**Table:**

Refinement on *F*^2^	Primary atom site location: structure-invariant direct methods
Least-squares matrix: full	Secondary atom site location: difference Fourier map
*R*[*F*^2^ > 2σ(*F*^2^)] = 0.045	Hydrogen site location: mixed
*wR*(*F*^2^) = 0.136	H atoms treated by a mixture of independent and constrained refinement
*S* = 1.06	*w* = 1/[σ^2^(*F*_o_^2^) + (0.0511*P*)^2^ + 0.8708*P*] where *P* = (*F*_o_^2^ + 2*F*_c_^2^)/3
2589 reflections	(Δ/σ)_max_ < 0.001
180 parameters	Δρ_max_ = 0.37 e Å^−^^3^
3 restraints	Δρ_min_ = −0.22 e Å^−^^3^

### Special details

**Table:**

Geometry. All e.s.d.'s (except the e.s.d. in the dihedral angle between two l.s. planes) are estimated using the full covariance matrix. The cell e.s.d.'s are taken into account individually in the estimation of e.s.d.'s in distances, angles and torsion angles; correlations between e.s.d.'s in cell parameters are only used when they are defined by crystal symmetry. An approximate (isotropic) treatment of cell e.s.d.'s is used for estimating e.s.d.'s involving l.s. planes.

### Fractional atomic coordinates and isotropic or equivalent isotropic displacement parameters (Å^2^)

**Table:**

	*x*	*y*	*z*	*U*_iso_*/*U*_eq_	
Mn1	0.5000	0.5000	0.5000	0.04895 (19)	
O4	0.3260 (2)	0.5819 (2)	0.52055 (10)	0.0539 (5)	
O3	0.5434 (2)	0.4939 (2)	0.59247 (10)	0.0565 (5)	
O1	0.7755 (2)	0.5192 (2)	0.62224 (11)	0.0632 (6)	
O2	0.8300 (3)	0.3560 (3)	0.56693 (12)	0.0734 (7)	
N2	0.4066 (3)	0.3381 (2)	0.52171 (12)	0.0512 (6)	
C1	0.9619 (3)	0.4152 (3)	0.64986 (13)	0.0518 (7)	
C2	0.9990 (3)	0.5096 (3)	0.68864 (13)	0.0516 (6)	
H2A	0.9520	0.5821	0.6899	0.062*	
N1	1.1449 (4)	0.5979 (4)	0.76146 (16)	0.0793 (10)	
C7	0.8479 (3)	0.4310 (3)	0.60979 (14)	0.0547 (7)	
C3	1.1051 (3)	0.4986 (4)	0.72582 (13)	0.0566 (7)	
C6	1.0307 (3)	0.3059 (4)	0.64803 (16)	0.0617 (8)	
H6	1.0069	0.2422	0.6218	0.074*	
C4	1.1723 (3)	0.3888 (4)	0.72398 (15)	0.0659 (9)	
H4A	1.2432	0.3795	0.7487	0.079*	
C11	0.2710 (3)	0.3669 (3)	0.53064 (17)	0.0618 (8)	
H11A	0.2348	0.3081	0.5592	0.074*	
H11B	0.2272	0.3586	0.4917	0.074*	
C5	1.1356 (3)	0.2928 (4)	0.68595 (16)	0.0682 (9)	
H5	1.1811	0.2193	0.6857	0.082*	
C10	0.2524 (4)	0.4967 (3)	0.55517 (17)	0.0657 (9)	
H10A	0.1640	0.5191	0.5525	0.079*	
H10B	0.2772	0.4999	0.5980	0.079*	
C9	0.4717 (4)	0.2849 (3)	0.57585 (18)	0.0690 (10)	
H9A	0.5494	0.2457	0.5628	0.083*	
H9B	0.4187	0.2222	0.5944	0.083*	
C8	0.5008 (4)	0.3832 (4)	0.62248 (16)	0.0724 (10)	
H8A	0.4259	0.4010	0.6464	0.087*	
H8B	0.5656	0.3537	0.6503	0.087*	
H4	0.276 (5)	0.606 (5)	0.484 (2)	0.099 (15)*	
H2	0.429 (4)	0.278 (3)	0.4912 (14)	0.071 (11)*	
H3	0.628 (6)	0.501 (5)	0.600 (3)	0.109 (18)*	
H1A	1.196 (5)	0.571 (6)	0.7962 (18)	0.13 (2)*	
H1B	1.087 (6)	0.664 (5)	0.771 (3)	0.18 (3)*	

### Atomic displacement parameters (Å^2^)

**Table:**

	*U*^11^	*U*^22^	*U*^33^	*U*^12^	*U*^13^	*U*^23^
Mn1	0.0487 (3)	0.0479 (3)	0.0503 (3)	−0.0033 (3)	−0.0032 (3)	−0.0027 (3)
O4	0.0536 (11)	0.0496 (11)	0.0586 (12)	0.0035 (10)	−0.0011 (10)	−0.0037 (10)
O3	0.0553 (12)	0.0658 (14)	0.0485 (10)	−0.0038 (11)	−0.0093 (9)	−0.0029 (11)
O1	0.0529 (12)	0.0758 (16)	0.0608 (12)	0.0082 (11)	−0.0109 (10)	−0.0144 (12)
O2	0.0762 (16)	0.0790 (17)	0.0650 (14)	0.0167 (14)	−0.0226 (12)	−0.0215 (13)
N2	0.0540 (14)	0.0457 (13)	0.0539 (13)	−0.0065 (11)	0.0018 (11)	−0.0061 (11)
C1	0.0494 (15)	0.0631 (18)	0.0429 (13)	−0.0042 (14)	0.0023 (12)	0.0026 (14)
C2	0.0465 (14)	0.0632 (17)	0.0452 (13)	−0.0017 (14)	0.0025 (11)	0.0061 (14)
N1	0.077 (2)	0.094 (3)	0.0674 (19)	−0.014 (2)	−0.0126 (16)	−0.0015 (19)
C7	0.0504 (16)	0.064 (2)	0.0496 (16)	−0.0016 (15)	−0.0012 (12)	−0.0024 (14)
C3	0.0520 (16)	0.073 (2)	0.0448 (13)	−0.0117 (16)	0.0003 (12)	0.0032 (16)
C6	0.067 (2)	0.065 (2)	0.0535 (16)	0.0017 (17)	−0.0015 (14)	−0.0011 (16)
C4	0.0520 (17)	0.094 (3)	0.0522 (17)	0.0043 (19)	−0.0040 (14)	0.0079 (18)
C11	0.0559 (19)	0.0569 (18)	0.073 (2)	−0.0107 (15)	0.0060 (15)	−0.0005 (16)
C5	0.063 (2)	0.079 (2)	0.0622 (19)	0.0150 (18)	−0.0014 (15)	0.0056 (18)
C10	0.0606 (19)	0.066 (2)	0.071 (2)	0.0055 (18)	0.0172 (16)	0.0018 (19)
C9	0.077 (2)	0.0563 (19)	0.074 (2)	−0.0046 (17)	−0.0092 (18)	0.0154 (17)
C8	0.076 (2)	0.088 (3)	0.0533 (18)	−0.014 (2)	−0.0086 (17)	0.0136 (19)

### Geometric parameters (Å, º)

**Table:**

Mn1—O3^i^	2.065 (2)	N1—C3	1.391 (5)
Mn1—O3	2.065 (2)	N1—H1A	0.97 (2)
Mn1—N2	2.067 (3)	N1—H1B	0.97 (2)
Mn1—N2^i^	2.068 (3)	C3—C4	1.387 (5)
Mn1—O4	2.096 (2)	C6—C5	1.393 (5)
Mn1—O4^i^	2.096 (2)	C6—H6	0.9300
O4—C10	1.424 (4)	C4—C5	1.384 (6)
O4—H4	0.99 (5)	C4—H4A	0.9300
O3—C8	1.438 (4)	C11—C10	1.515 (5)
O3—H3	0.92 (6)	C11—H11A	0.9700
O1—C7	1.255 (4)	C11—H11B	0.9700
O2—C7	1.251 (4)	C5—H5	0.9300
N2—C9	1.482 (4)	C10—H10A	0.9700
N2—C11	1.485 (4)	C10—H10B	0.9700
N2—H2	0.959 (19)	C9—C8	1.502 (5)
C1—C2	1.383 (5)	C9—H9A	0.9700
C1—C6	1.390 (5)	C9—H9B	0.9700
C1—C7	1.501 (4)	C8—H8A	0.9700
C2—C3	1.391 (4)	C8—H8B	0.9700
C2—H2A	0.9300		
			
O3^i^—Mn1—O3	180.00 (14)	O1—C7—C1	117.0 (3)
O3^i^—Mn1—N2	98.21 (10)	C4—C3—N1	121.5 (3)
O3—Mn1—N2	81.79 (10)	C4—C3—C2	118.2 (3)
O3^i^—Mn1—N2^i^	81.79 (10)	N1—C3—C2	120.3 (4)
O3—Mn1—N2^i^	98.21 (10)	C1—C6—C5	119.3 (4)
N2—Mn1—N2^i^	180.0	C1—C6—H6	120.4
O3^i^—Mn1—O4	89.88 (9)	C5—C6—H6	120.4
O3—Mn1—O4	90.12 (9)	C5—C4—C3	121.1 (3)
N2—Mn1—O4	83.54 (10)	C5—C4—H4A	119.5
N2^i^—Mn1—O4	96.46 (10)	C3—C4—H4A	119.5
O3^i^—Mn1—O4^i^	90.11 (9)	N2—C11—C10	111.6 (3)
O3—Mn1—O4^i^	89.89 (9)	N2—C11—H11A	109.3
N2—Mn1—O4^i^	96.47 (10)	C10—C11—H11A	109.3
N2^i^—Mn1—O4^i^	83.53 (10)	N2—C11—H11B	109.3
O4—Mn1—O4^i^	180.0	C10—C11—H11B	109.3
C10—O4—Mn1	108.80 (19)	H11A—C11—H11B	108.0
C10—O4—H4	107 (3)	C4—C5—C6	120.2 (4)
Mn1—O4—H4	115 (3)	C4—C5—H5	119.9
C8—O3—Mn1	113.5 (2)	C6—C5—H5	119.9
C8—O3—H3	108 (3)	O4—C10—C11	110.0 (3)
Mn1—O3—H3	113 (4)	O4—C10—H10A	109.7
C9—N2—C11	115.4 (3)	C11—C10—H10A	109.7
C9—N2—Mn1	106.7 (2)	O4—C10—H10B	109.7
C11—N2—Mn1	108.5 (2)	C11—C10—H10B	109.7
C9—N2—H2	100 (2)	H10A—C10—H10B	108.2
C11—N2—H2	118 (3)	N2—C9—C8	110.9 (3)
Mn1—N2—H2	108 (2)	N2—C9—H9A	109.4
C2—C1—C6	119.8 (3)	C8—C9—H9A	109.4
C2—C1—C7	120.0 (3)	N2—C9—H9B	109.4
C6—C1—C7	120.2 (3)	C8—C9—H9B	109.4
C1—C2—C3	121.5 (3)	H9A—C9—H9B	108.0
C1—C2—H2A	119.3	O3—C8—C9	110.4 (3)
C3—C2—H2A	119.3	O3—C8—H8A	109.6
C3—N1—H1A	112 (4)	C9—C8—H8A	109.6
C3—N1—H1B	119 (5)	O3—C8—H8B	109.6
H1A—N1—H1B	114 (5)	C9—C8—H8B	109.6
O2—C7—O1	124.2 (3)	H8A—C8—H8B	108.1
O2—C7—C1	118.8 (3)		
			
C6—C1—C2—C3	0.7 (5)	C2—C3—C4—C5	0.4 (5)
C7—C1—C2—C3	−179.2 (3)	C9—N2—C11—C10	88.9 (4)
C2—C1—C7—O2	166.2 (3)	Mn1—N2—C11—C10	−30.7 (3)
C6—C1—C7—O2	−13.6 (5)	C3—C4—C5—C6	0.9 (6)
C2—C1—C7—O1	−14.4 (5)	C1—C6—C5—C4	−1.4 (5)
C6—C1—C7—O1	165.7 (3)	Mn1—O4—C10—C11	−38.9 (3)
C1—C2—C3—C4	−1.2 (5)	N2—C11—C10—O4	47.5 (4)
C1—C2—C3—N1	175.9 (3)	C11—N2—C9—C8	−76.9 (4)
C2—C1—C6—C5	0.7 (5)	Mn1—N2—C9—C8	43.7 (4)
C7—C1—C6—C5	−179.5 (3)	Mn1—O3—C8—C9	17.4 (4)
N1—C3—C4—C5	−176.6 (3)	N2—C9—C8—O3	−40.8 (4)

Symmetry code: (i) −*x*+1, −*y*+1, −*z*+1.

### Hydrogen-bond geometry (Å, º)

**Table:**

*D*—H···*A*	*D*—H	H···*A*	*D*···*A*	*D*—H···*A*
N2—H2···O2^ii^	0.96 (3)	2.19 (3)	2.965 (4)	137 (3)
N1—H1*A*···O1^iii^	0.97 (2)	2.05 (2)	3.008 (4)	170 (5)
O4—H4···O2^i^	0.99 (5)	1.63 (5)	2.611 (3)	169 (4)
O3—H3···O1	0.92 (6)	1.65 (6)	2.562 (3)	173 (5)

Symmetry codes: (i) −*x*+1, −*y*+1, −*z*+1; (ii) *x*−1/2, −*y*+1/2, −*z*+1; (iii) *x*+1/2, *y*, −*z*+3/2.

## References

[bb1] Ashurov, J. M., Ibragimov, A. B. & Ibragimov, B. T. (2015). *Polyhedron*, **102**, 441–446.

[bb2] Bertrand, J. A., Fujita, E. & VanDerveer, D. G. (1979). *Inorg. Chem.* **18**, 230–233.

[bb3] Buvaylo, E. A., Kokozay, V. N., Vassilyeva, O. Yu., Skelton, B. W. & Jezierska, J. (2009). *Inorg. Chim. Acta*, **362**, 2429–2434.

[bb4] Fang, Z. & Nie, Q. (2011). *J. Coord. Chem.* **64**, 2573–2582.

[bb5] Flemig, H., Pantenburg, I. & Meyer, G. (2008). *J. Alloys Compd.* **451**, 429–432.

[bb6] Gao, J., Wang, J. & Nie, J. (2011). *Acta Cryst.* C**67**, m181–m184. 10.1107/S0108270111017690 21633150

[bb7] Groom, C. R. & Allen, F. H. (2014). *Angew. Chem. Int. Ed.* **53**, 662–671. 10.1002/anie.201306438 24382699

[bb8] Kozioł, A. E., Klimek, B., Stępniak, K., Rzączyńska, Z., Brzvska, W., Bodak, O. I., Akselrud, L. G., Pavlyuk, V. V. & Tafeenko, V. A. (1992). *Z. Kristallogr.* **200**, 25–33.

[bb9] Macrae, C. F., Edgington, P. R., McCabe, P., Pidcock, E., Shields, G. P., Taylor, R., Towler, M. & van de Streek, J. (2006). *J. Appl. Cryst.* **39**, 453–457.

[bb10] Murugavel, R. & Banerjee, S. (2003). *Inorg. Chem. Commun.* **6**, 810–814.

[bb11] Oxford Diffraction (2009). *CrysAlis PRO*. Oxford Diffraction Ltd, Yarnton, England.

[bb12] Ozhafarov, N. Kh., Amiraslanov, I. R., Nadzhafov, G. N., Movsumov, E. M. & Mamedov, Kh. S. (1981). *Zh. Strukt. Khim.* **22**, 121–122.

[bb13] Palanisami, N., Rajakannu, P. & Murugavel, R. (2013). *Inorg. Chim. Acta*, **405**, 522–531.

[bb14] Petrović, Z. D., Djuran, M. I., Heinemann, F. W., Rajković, S. & Trifunović, S. R. (2006). *Bioorg. Chem.* **34**, 225–234. 10.1016/j.bioorg.2006.06.003 16889816

[bb15] Sheldrick, G. M. (2008). *Acta Cryst.* A**64**, 112–122. 10.1107/S0108767307043930 18156677

[bb16] Sheldrick, G. M. (2015). *Acta Cryst.* C**71**, 3–8.

[bb17] Shen, F. M. & Lush, S. F. (2010). *Acta Cryst.* E**66**, m1427. 10.1107/S1600536810041322 PMC3009093 21588853

[bb18] Smith, G., Cloutt, B. A., Lynch, D. E., Byriel, K. A. & Kennard, C. H. L. (1998). *Inorg. Chem.* **37**, 3236–3242.

[bb19] Sundberg, M. R., Koskimies, J. K., Matikainen, J. & Tylli, H. (1998). *Inorg. Chim. Acta*, **268**, 21–30.

[bb20] Tan, A.-Z., Wei, Y.-H., Chen, Z.-L., Liang, F.-P. & Hu, R.-X. (2006). *Wuji Huaxue Xuebao*, **22**, 394–398.

[bb21] Tsaryuk, V., Vologzhanina, A., Zhuravlev, K., Kudryashova, V., Szostak, R. & Zolin, V. (2014). *J. Photochem. Photobiol. A*, **285**, 52–61.

[bb22] Wang, R., Hong, M., Luo, J., Jiang, F., Han, L., Lin, Z. & Cao, R. (2004). *Inorg. Chim. Acta*, **357**, 103–114.

[bb23] Wang, R., Yuan, D., Jiang, F., Han, L., Gao, S. & Hong, M. (2006). *Eur. J. Inorg. Chem.* pp. 1649–1656.

[bb24] Wei, Y.-H., Tan, A.-Z., Chen, Z.-L., Liang, F., -, P. & Hu, R.-X. (2006). *Jiegou Huaxue*, **25**, 343–348.

[bb25] Yilmaz, V. T., Karadag, A., Thöne, C. & Herbst-Irmer, R. (2000). *Acta Cryst.* C**56**, 948–949. 10.1107/s0108270100007289 10944284

[bb26] Zhang, W.-Z. (2006). *Acta Cryst.* E**62**, m857–m859.

